# Resolution of severe dilated cardiomyopathy with significant arrhythmia burden using hydroquinidine, in addition to guideline-directed medical therapy, in a patient with a pathogenic *SCN5A* variant: a case report

**DOI:** 10.1093/ehjcr/ytag100

**Published:** 2026-02-06

**Authors:** Rebecca L M Griffiths, Peter J Cowburn, Catherine Mercer, Michael Papadakis, Elijah R Behr

**Affiliations:** Cardiovascular and Genomics Research Institute, School of Health and Medical Sciences, City St George’s, University of London, Tooting Campus, Cranmer Terrace, London SW17 0RE, United Kingdom; Department of Cardiology, Southampton General Hospital, Tremona Road, Southampton, Hampshire SO16 6YD, United Kingdom; Department of Genetics, Southampton General Hospital, Tremona Road, Southampton, Hampshire SO16 6YD, United Kingdom; Cardiovascular and Genomics Research Institute, School of Health and Medical Sciences, City St George’s, University of London, Tooting Campus, Cranmer Terrace, London SW17 0RE, United Kingdom; Cardiovascular and Genomics Research Institute, School of Health and Medical Sciences, City St George’s, University of London, Tooting Campus, Cranmer Terrace, London SW17 0RE, United Kingdom

**Keywords:** Dilated cardiomyopathy, Arrhythmogenic cardiomyopathy, *SCN5A*, p.R814W, Hydroquinidine, LifeVest, Case report

## Abstract

**Background:**

Dilated cardiomyopathy has a diverse aetiology. Around 20% of cases have an underlying genetic cause. A subset of patients with dilated cardiomyopathy is prone to arrhythmia (‘arrhythmogenic’ cardiomyopathy). (Likely) Pathogenic variants of *SCN5A*, the gene coding for the alpha subunit of the main cardiac sodium voltage-gated channel, are a known cause of this subset.

**Case summary:**

A 17-year-old male presents with new-onset severe left ventricular systolic dysfunction with atrial flutter and significant ventricular ectopy. Despite medical therapy, his management was challenging. A LifeVest was fitted to allow outpatient optimization of his medications whilst bridging to a decision about implantable cardioverter defibrillator implantation. Specialist genetic testing revealed a pathogenic variant in *SCN5A* (p.R814W) leading to gain of function. This prompted the use of a sodium channel blocker, hydroquinidine. Hydroquinidine resulted in complete resolution of arrhythmia and improvement of ventricular size and function. Its effect was confirmed on accidental withdrawal of hydroquinidine due to supply issues, resulting in recurrence of atrial arrhythmia.

**Discussion:**

This atypical presentation of a cardiomyopathy was driven, at least in part, by the patient’s extensive arrhythmia. Previous research has shown variable, short-term effects of sodium channel antagonists in familial p.R814W variants. Contrastingly, in our patient, sustained and long-term improvement was observed with the use of hydroquinidine. Close multidisciplinary team working and early genetic testing facilitated personalized care in our patient’s case, resulting in a favourable outcome.

Learning points

- Early genetic testing was key for guiding management in this atypical presentation of early cardiomyopathy, predominantly driven by tachyarrhythmia.
- Close multidisciplinary team working between specialists was vital to produce a favourable outcome.
- The early use of a LifeVest ensured a safe discharge, allowing medications to be optimized and a decision to be made regarding ICD implantation in this young patient.

## Introduction

Dilated cardiomyopathy (DCM) is diagnosed in patients with dilatation of their left ventricle (LV, defined as an LV end-diastolic diameter, EDD, >58 mm in males and >52 in females) and regional/global LV systolic impairment (defined as an LV ejection fraction, EF, <50%) that cannot be explained by loading conditions or coronary artery disease alone.^1,2^ In around 20% of cases, (likely) pathogenic genetic variants are responsible for its development.^3^

A subgroup of patients with DCM is predisposed to arrhythmias, so-called ‘arrhythmogenic’ cardiomyopathy. Several genes have been implicated in the pathophysiology of this phenotype, including *SCN5A*, the gene coding for the alpha subunit of the primary cardiac sodium voltage-gated channel, Na_V_1.5.^4^ We present the case of a 17-year-old gentleman with severe DCM and significant arrhythmic burden and demonstrate how early genetic testing personalized his care and produced an optimal clinical outcome.

## Summary figure

**Table:** 

13/11/2019	Presentation to local district general hospital with heart failure and atrial arrhythmias
15/11/2019	Transoesophageal echocardiogram-guided direct current cardioversion (DCCV)
21/11/2019	Repeat DCCV on amiodarone
04/12/2019	Discharged with LifeVest (bisoprolol withheld due concerns about junctional rhythm)
23/12/2019	Bisoprolol reintroduced with ongoing uptitration of medical therapy
09/03/2020	Sinus rhythm with left ventricular (LV) improvement on optimal medical therapy Amiodarone stopped
04/06/2020	Holter monitor (off amiodarone)—very frequent atrial/ventricular ectopy and paroxysmal atrial flutter
06/07/2020	Pathogenic variant in *SCN5A* gene detected c.2440C>T (p.Arg814Trp), R814W
15/07/2020	Reviewed at St George’s Hospital—decision made to proceed with transvenous implantable cardioverter defibrillation (ICD) implantation with a trial of hydroquinidine
21/08/2020	Admitted for ICD and commenced hydroquinidine
20/04/2021	LV function improved, ectopics suppressed, NT-proBNP normalized
04/01/2024	Asymptomatic LV dilatation resolved, function only mildly reduced
18/06/2024	Device alert, frequent atrial arrhythmias—the patient had run out of hydroquinidine (difficulty sourcing medication)
26/07/2024	Repeat download 1 month following recommencement of hydroquinidine—no atrial arrhythmias detected

## Case presentation

A 17-year-old-male presented to his GP with increasing breathlessness, cough, palpitations, and chest pain. He had experienced a viral illness 1 week previously. He had no underlying medical problems and played county rugby. There was no family history of cardiac disease. He took no regular medication. He did not smoke, drank little alcohol, and took no recreational drugs. He was referred to his local hospital for further assessment.

On arrival, his vital signs indicated: an irregular, tachycardic pulse (130 b.p.m.); a low blood pressure (98/61 mmHg); and a raised respiratory rate (40/min) with oxygen saturations of 92% on air. He was afebrile.

**Figure 1:**
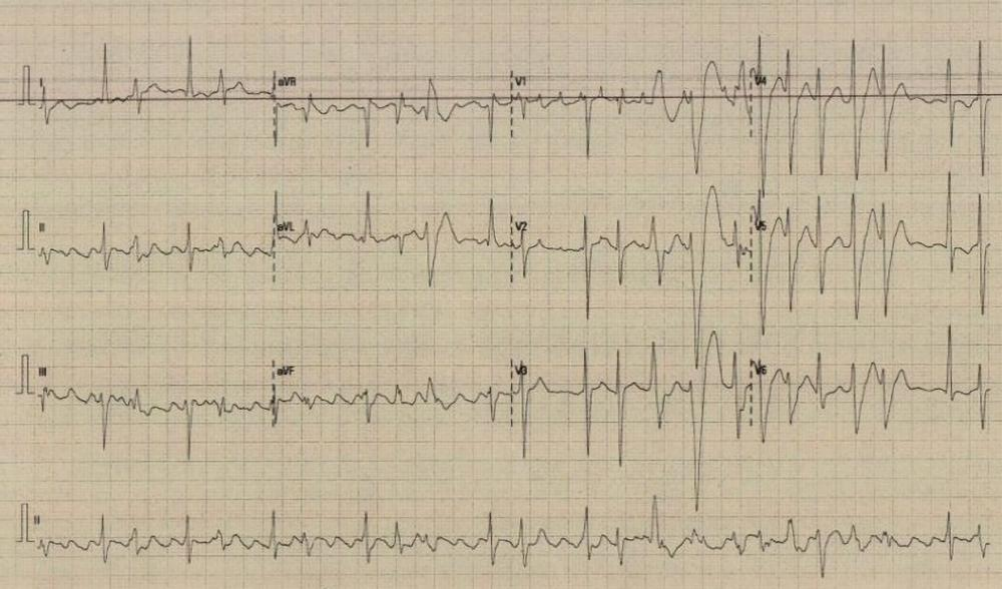
The patient’s presenting electrocardiogram demonstrating typical atrial flutter and frequent multifocal ventricular ectopy.

Blood tests revealed a leucocytosis, deranged liver enzymes, and a normal C-reactive protein and troponin T. The patient’s presenting electrocardiogram (ECG) is shown in *Figure 1*. Heart rhythm monitoring revealed very frequent ventricular ectopy and short runs of non-sustained ventricular tachycardia. A chest X-ray showed cardiomegaly with mild pulmonary congestion. Bedside echocardiography revealed severe LV systolic dysfunction with elevated right ventricular systolic pressure.

**Figure 2:**
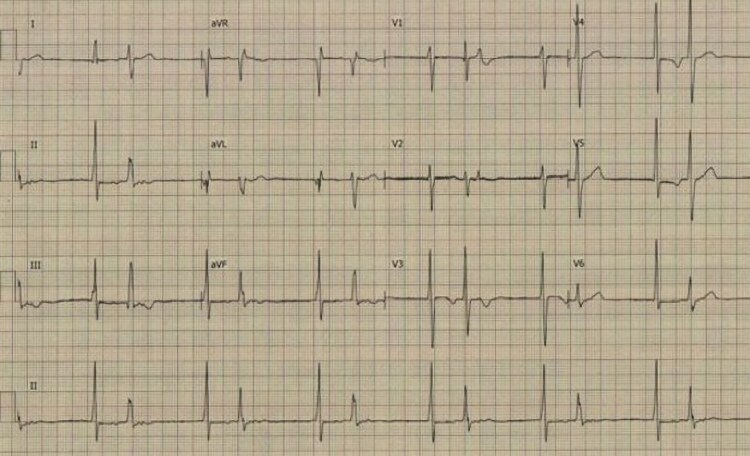
The patient’s electrocardiogram following transoesophageal echocardiogram-guided direct current cardioversion demonstrating junctional rhythm with bigeminy.

Despite commencing appropriate medications for heart failure and atrial flutter, the patient did not improve. A transoesophageal echocardiogram-guided DC cardioversion (DCCV) was therefore performed, and his subsequent ECG is shown in *Figure 2*. Bisoprolol was uptitrated and amiodarone commenced for suppression of arrhythmia. He was transferred to his local tertiary cardiology centre for further management.

A cardiac MRI (CMR) on arrival confirmed significant LV dilatation and severely reduced function (see Supplementary material online, *Videos S1–S3*). There was no inflammation, myocardial oedema, or late enhancement (see Supplementary material online, *Figures S1*–*S3*).

**Figure 3:**
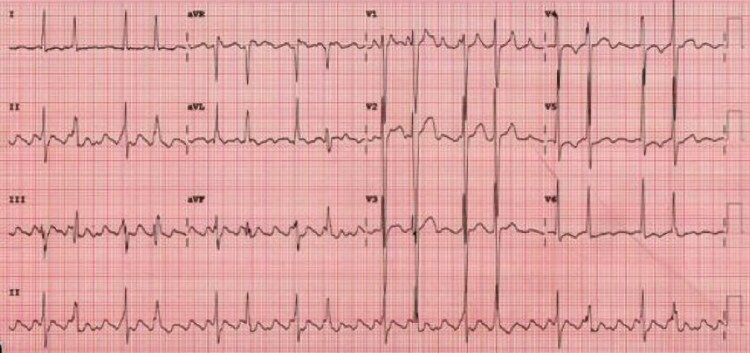
The patient’s electrocardiogram demonstrating recurrence of atrial tachyarrhythmia and frequent ectopy following bisoprolol withdrawal.

A specialist genetics review was requested and a dilated and arrhythmogenic cardiomyopathy gene panel sent (see *Appendix 1* for the list of genes assessed). He commenced regular furosemide; eplerenone and amiodarone were continued; and sacubitril/valsartan initiated. Bisoprolol was stopped due to junctional rhythm, with a subsequent relapse of atrial tachyarrhythmia (*Figure 3*). Direct current cardioversion was repeated leading to restoration of a junctional rhythm with bigeminy, as shown previously (*Figure 2*).

The patient improved, and prior to discharge, a cardiopulmonary exercise test was performed. He achieved a peak workload of 216 W with a peak heart rate and blood pressure of 148 b.p.m. and 152/80 mmHg, respectively. Low-level exercise induced frequent ventricular ectopy and bigeminy, settling at peak exercise but returning quickly in recovery. His exercise capacity was severely impaired (VO_2_peak 16.9 mL/min/kg, 34% of predicted) by his cardiac disease evidenced by a lack of rise in oxygen pulse at higher exercise intensities. The anaerobic threshold was 11.4 mL/min/kg. A multidisciplinary team discussion concluded that the patient’s arrhythmic risk was high, and he should be discharged with a LifeVest whilst his medications were optimized and ICD implantation considered.

The patient continued to improve symptomatically with medication optimization and gradual reintroduction of bisoprolol. This improvement was reflected in his investigations (resolution of ectopy on ECG, improvement of ventricular size and function on echocardiography and CMR). Amiodarone was stopped (in view of its long-term adverse effects) and the LifeVest retained.

A follow-up Holter off amiodarone showed a resurgence of arrhythmia with frequent junctional and ventricular ectopy, episodes of bigeminy, and periods of atrial flutter with very little sustained normal sinus rhythm. His clinical picture was therefore one of DCM attributable, at least in part, to atrial, Purkinje, and ventricular arrhythmias.

The patient’s genetic test revealed a pathogenic (Class V) variant in *SCN5A* (c.2440C>T (p.Arg814Trp)), p.R814W. He was thereafter referred for specialist input. Subsequently, he was admitted in August 2020 for introduction of oral hydroquinidine (300 mg BD Modified-Release) and implantation of a transvenous ICD.

The patient was a keen sportsman prior to diagnosis and was therefore reviewed by a sports cardiology specialist. He was advised to avoid high-intensity competitive sport but encouraged to exercise to maintain physical fitness and mental well-being. His school was written to with specifics of what activities were reasonable.

**Figure 4:**
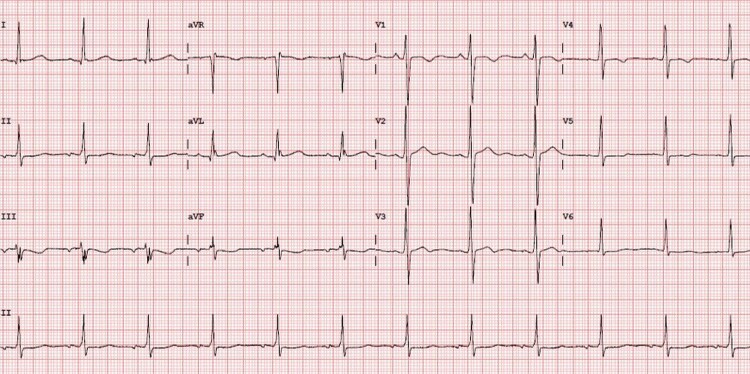
The patient’s electrocardiogram whilst taking hydroquinidine. Note the absence of ectopy or atrial arrhythmia.

**Figure 5:**
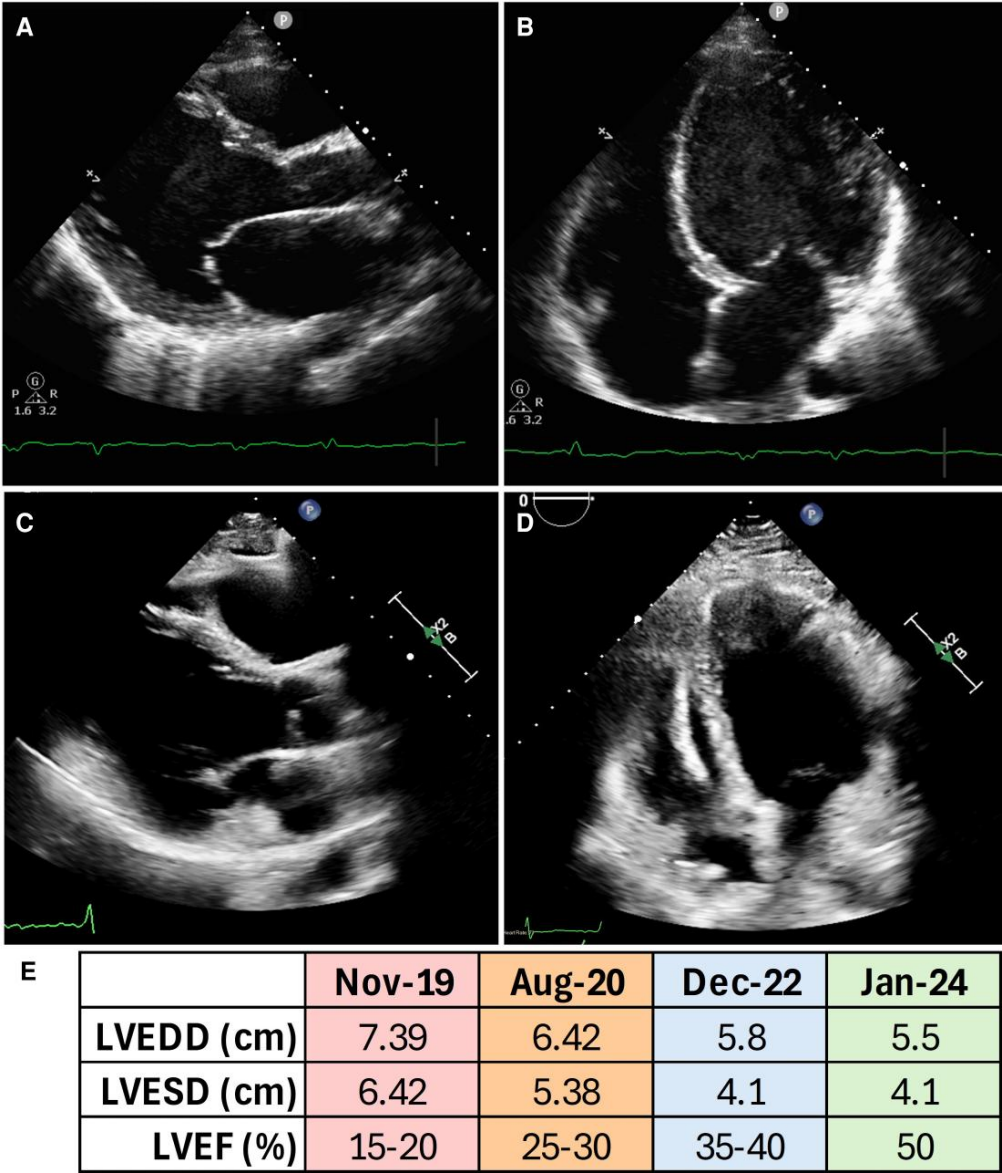
Visual and numeric demonstration of the improvement of the patient’s left ventricular structure and function over time. Parasternal long axis (*A* and *C*) and apical four-chamber (*B* and *D*) views from the patient’s initial and most recent echocardiograms, performed in November 2019 (*A* and *B*) and January 2024 (*C* and *D*), respectively. There is clear visual improvement in left ventricular dilatation. Panel (*E*) presents measurements of the patient’s left ventricular end-diastolic diameter, left ventricular end-systolic diameter, and left ventricular ejection fraction from serial imaging. An improvement of these parameters is observed over time.

Subsequent ECG and Holter monitoring on hydroquinidine showed neither arrhythmia nor ectopy (*Figure 4*). His latest echocardiogram showed resolution of ventricular dilatation with only mild LV impairment (EF 50%; *Figure 5*).

In May 2024 due to a misunderstanding about how much medication he had remaining, the patient ran out of hydroquinidine and remained off this medication for 3 weeks due to supply issues. His other medications continued. An ICD download reported 35 episodes of atrial tachyarrhythmia with a maximum duration of 58 min, correlating to symptoms of weakness and feeling ‘jittery’. On resumption of hydroquinidine, his symptoms improved and the atrial tachyarrhythmias resolved, with none reported on his latest ICD download.

## Discussion

Early genetic testing in our patient’s case facilitated the use of a personalized genomics-based therapy to produce a favourable clinical response. The p.R814W variant in *SCN5A* has been observed both *de novo*, through analysis of probands within a DCM cohort, as well as in a family with a history of sudden cardiac death and DCM.^4,5^ Of note, in our case, the p.R814W variant was not detected in our patient’s parents, suggesting a *de novo* finding. A group in Chicago, USA, have also shown that the p.R814W variant co-segregates with DCM, sudden death, and the need for transplantation.

The variant affects the fourth, voltage-sensing segment (S4) of the second domain of Na_v_1.5 with replacement of a conserved arginine residue with tryptophan.^6^ The p.R814W variant predominantly causes gain of function through mechanisms of altered activation, increase in window current, and slower deactivation of Na_v_1.5, resulting in enhanced sodium current and supraventricular and ventricular hyperexcitability, thereby causing frequent arrhythmia.^5,6^ Contractile impairment may result from this frequent arrhythmia, or through other mechanisms such as blunting/reversal of the force–frequency relationship, abnormalities in intracellular calcium levels (caused by a higher intracellular sodium), ineffective regulation of pH (caused by abnormal sodium and calcium homeostasis), and effects on mitochondria (e.g. swelling and dysfunction, thereby leading to metabolic disruption of cardiomyocytes).^5–7^ The variant’s functional effect justified use of hydroquinidine, a sodium channel antagonist leading to a striking improvement in LV size and function, as well as his arrhythmic burden.

Improvement of LV dynamics and arrhythmias with quinidine monotherapy has previously been observed in *SCN5A* gain-of-function variants.^8–10^ The p.R222Q *SCN5A* variant which, like p.R814W, is in a voltage-sensing segment of Na_V_1.5 was shown in a case report to reduce arrhythmia and improve LV function.^8^ Of note, unlike in the present case, complete resolution of arrhythmia and restoration of LV function was not observed.^8^ This may be due to the different biophysical effects of disparate *SCN5A* variants, a shorter follow-up period of the case (2.5 years), dose of quinidine used, or due to confounding effects from concurrent coronary artery disease.^8^

Zakrzewska-Koperska *et al*.^5^ studied a family of patients with arrhythmogenic DCM and the p.R814W variant. Interestingly, quinidine monotherapy was met with mixed success. Combinations of Class I antiarrhythmics were required to restore clinical stability in this family.^5^ Furthermore, the follow-up period in this case series was relatively short (1.5 years) meaning sustained improvement could not be fairly assessed. Contrastingly, in our case, hydroquinidine monotherapy led to sustained improvement of symptoms, LV size and function, and arrhythmic burden over a 4-year follow-up period—to the authors’ knowledge, the first description of such.

Recent ESC guidelines on the management of cardiomyopathies and ventricular arrhythmias recommend that genetic testing is undertaken in patients with cardiomyopathies where genetics will help to establish a diagnosis, prognosticate, stratify therapy or assist in reproductive management (Class IB Level of Evidence).^2,11^ In our patient’s case, prompt genetic testing facilitated the use of personalized therapy, producing an optimal and sustained clinical improvement.

## Lead author biography


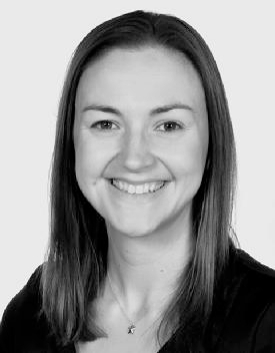


Rebecca graduated from Cardiff University Medical School in 2017, having previously achieved a First Class Honours in her Intercalated BSc in Biomedical Sciences (Neuroscience). As an undergraduate, she developed a strong interest in cardiology, later earning a distinction in an MSc in Translational Cardiovascular Medicine at the University of Bristol. Her academic training, combined with experience as a cardiology registrar, prompted a focused interest in inherited cardiac conditions. She currently works a Clinical Research Fellow at City St George’s, University of London, and is undertaking a PhD focussing on the inherited arrhythmia syndrome, Brugada Syndrome.

## Supplementary Material

ytag100_Supplementary_Data

## Data Availability

Additional non-identifiable electrocardiographic and imaging data are available upon request.

## Notes

**Consent:** The authors confirm that the written consent for submission and publication of this case report, including images and associated text, has been obtained from the patient in line with COPE guidance.

## Appendix 1

**Table:** Table listing the genes included on the dilated and arrhythmogenic cardiomyopathy gene panel that was sent for the patient. Gene symbols and names are provided.

Symbol	Gene name
*ACTC1*	Actin alpha cardiac muscle 1
*ACTN2*	Actinin alpha 2
*BAG3*	BAG cochaperone 3
*CDH2*	Cadherin 2
*DES*	Desmin
*DMD*	Dystrophin
*DOLK*	Dolichol kinase
*DSC2*	Desmocollin 2
*DSG2*	Desmogelin 2
*DSP*	Desmoplakin
*EMD*	Emerin
*FLNC*	Filamin C
*JUP*	Junction plakoglobin
*LAMP2*	Lysosomal associated membrane protein 2
*LMNA*	Lamin A/C
*MYBPC3*	Myosin-binding protein C3
*MYH7*	Myosin heavy chain 7
*NEXN*	Nexilin F-actin binding protein
*NKX2-5*	NK2 homeobox 5
*PKP2*	Plakophilin 2
*PLN*	Phospholamban
*RBM20*	RNA-binding motif protein 20
*RYR2*	Ryanodine receptor 2
*SCN5A*	Sodium voltage-gated channel alpha subunit 5
*TMEM43*	Transmembrane protein 43
*TNNC1*	Troponin C1, slow skeletal and cardiac type
*TNNI3*	Troponin I3, cardiac type
*TNNI3K*	TNNI3-interacting kinase
*TNNT2*	Troponin T2, cardiac type
*TPM1*	Tropomyosin 1
*TTN*	Titin
*VCL*	Vinculin
