# Supplementary figures and images for: Resolution of severe dilated cardiomyopathy with significant arrhythmia burden using hydroquinidine, in addition to guideline-directed medical therapy, in a patient with a pathogenic SCN5A variant: a case report
Source: Eur Heart J Case Rep. 2026 Feb 6;10(3):ytag100. doi: 10.1093/ehjcr/ytag100 (PMC12952205; doi:10.1093/ehjcr/ytag100)

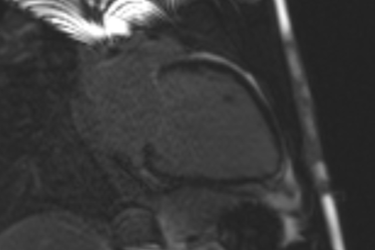

Supplement: ytag100_Supplementary_Data [file ytag100_supplementary_data.zip › Figure S1 - Gadolinium - 2 Chamber View.png]

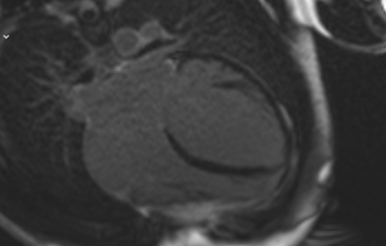

Supplement: ytag100_Supplementary_Data [file ytag100_supplementary_data.zip › Figure S2 - Gadolinium - 4 Chamber View.png]

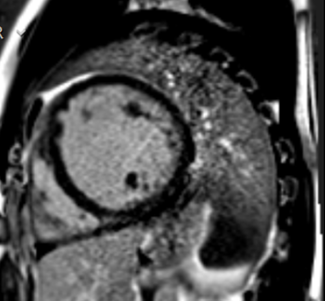

Supplement: ytag100_Supplementary_Data [file ytag100_supplementary_data.zip › Figure S3 - Gadolinium - Short Axis View.png]
